# Interventions to Increase the Uptake of Mammography amongst Low Income Women: A Systematic Review and Meta-Analysis

**DOI:** 10.1371/journal.pone.0055574

**Published:** 2013-02-22

**Authors:** Michael P. Gardner, Abbey Adams, Mona Jeffreys

**Affiliations:** 1 School of Social and Community Medicine, University of Bristol, Bristol, United Kingdom; 2 University of Bristol, Bristol, United Kingdom; Harvard Medical School, United States of America

## Abstract

**Background:**

Two previous reviews found that access-enhancing interventions were effective in increasing mammography uptake amongst low-income women. The purpose of this study was to estimate the magnitude of the effect of interventions used to increase uptake of mammography amongst low-income women.

**Methods:**

Searches were conducted in MEDLINE and EMBASE (2002–April 2012) using relevant MeSH terms and keywords. Randomised controlled trials which aimed to increase mammography use in an asymptomatic low-income population and which had as an outcome receipt of a mammogram, were eligible for inclusion. The primary outcome was the post-intervention difference in the proportion of women who had a mammogram in the intervention and control groups. The quality of the studies was assessed using the Cochrane risk of bias tool. We calculated summary estimates using random effects meta-analyses. Possible reasons for heterogeneity were investigated using sub-group analyses and meta-regression. Publication bias was assessed using Egger's test.

**Results:**

Twenty-one studies met the inclusion criteria, including 33 comparisons. Interventions increased the uptake of mammography in low income women by an additional 8.9% (95% CI 7.3 to 10.4%) compared to the control group. There was some evidence that interventions with multiple strategies were more effective than those with single strategies (p  = 0.03). There was some suggestion of publication bias. The quality of the included studies was often unclear. Omitting those with high risk of bias has little effect on the results.

**Conclusions:**

Interventions can increase mammography uptake among low-income women, multiple interventions being the most effective strategy. Given the robustness of the results to sensitivity analyses, the results are likely to be reliable. The generalisability of the results beyond the US is unclear.

## Introduction

Breast cancer is the most common female cancer in developed countries [1]. There is a substantial body of evidence that shows the earlier breast cancer is detected the greater the chance of survival [2], [3]. In the EUROCARE-4 (1995–1999) study [4], 5-year relative survival varied between 23 European countries, ranging from 69.2% in the Czech Republic to 88.0% in Iceland, with the figure for the UK close to the mean at 77%. A main determinant of low survival in breast cancer is advanced stage at diagnosis [5]. Mammography is widely accepted as a useful and feasible way of detecting early malignancies; although there are ongoing discussions regarding the effectiveness of mammographic screening, relating to false positive rates and over-diagnosis [3], [6]. We have recently demonstrated that about two thirds of the inequalities in 5-year breast cancer survival in South West England are attributable to non-attendance at screening [7]. The potential impact of increasing screening attendance on inequalities in breast cancer outcomes is therefore considerable.

Recent studies have shown that the rates of mammography utilisation have steadily been increasing [8]. However, women from ethnic minorities, older women and women of low-income in the UK [9] and the US [10]–[12] are less likely to attend mammography.

Although interventions to increase attendance at mammography have been evaluated [13], the increases do not always occur equally. Women with historically low rates of screening do not necessarily benefit from interventions offered on a population level; indeed, if interventions work better in improving attendance amongst higher income women there is a risk that those interventions could increase inequalities [14].

The aim of this study was to synthesise recent randomised controlled trials (RCTs) which aimed to increase mammography amongst women of low-income. A previous systematic review and meta-analysis [15] found that access-enhancing interventions were the most effective. Another systematic review [16] concluded that interventions which used peer educators, or were access-enhancing or which used multi-component strategies were effective in increasing mammography amongst low-income women. This review is intended to update these publications with more rigorous methodology, including meta-analyses, exploration of sources of heterogeneity and quality assessment. The specific objectives are to synthesise the recent efforts to increase uptake of mammography amongst low-income women and to try to identify which intervention characteristics contributed to how effective they were.

## Methods

We undertook a systematic review of the published literature, using methods following the PRISMA statement [17].

### Search strategy

A search strategy using text and exploded MeSH terms (Table s1) was developed by two authors. Using this, searches of MEDLINE and EMBASE for articles published from 2002 to April 2012 were performed. An additional hand search was performed of the bibliographies of relevant studies identified from the computerised search. We also contacted the author of a conference publication to ascertain if the study had been published in a peer reviewed journal.

### Inclusion criteria

To be included a study had to a) state that the study aimed to increase mammography use in an asymptomatic low-income population b) have as an outcome receipt of mammogram (rather than intention to attend mammography) either by self-report or medical records and c) be a RCT published in full. No language restriction was applied to the search. Studies which tested single or multiple interventions were included. If the target population was chosen based on ethnicity then we checked the income data in the paper and included the study if the participants were from a low-income population. If there was no income data then we excluded the study. We excluded studies where randomisation was on a county or area level but included those randomised on small group level, e.g. church. Titles and abstracts and if necessary, full text articles were independently reviewed by two authors (MG, MJ). In cases of uncertainty, eligibility was decided through discussion. A protocol does not exist for the systematic review.

### Data extraction

Data extraction tables were developed and modified following discussions between two reviewers prior to extraction of data. Relevant participant, intervention, control and outcome data were extracted, see Tables 1a and 1b. When more than one length of follow-up was extracted per study, the longer follow-up length was included in the analysis. Data extraction was performed by two reviewers and differences were resolved through discussion. Interventions were classified as ‘simple’ (e.g. a letter, phone call, video, computer-assisted instruction, print media (including tailored magazines) or access to free mammography); ‘face to face’ (e.g. an education session or home visit) or ‘multiple’ (multi-component interventions e.g. phone call and letter). Time lag was defined as the time from the end of the intervention to the date of publication. The primary outcome was defined as the difference in the proportion of women who had had a mammogram by the end of the follow-up period between the intervention and control group (Table 2).

**Table 1:** Description of studies included in the review.

Reference	Source of participants	Screening status of participants	Description of intervention	Control group treatment	Period of intervention	Additional Details
Ahmed, 2010 [51]	Managed Care Organisations	Non-compliant in previous 2–3 years.	Simple- Reminder letter Multiple- Two reminder letters and counselling	Usual care	1999-2001	Free mammography for eligible members.
Champion, 2006 [30]	Clinics, churches and low income housing associations	No mammogram in the previous 18 months.	Simple- Culturally appropriate video; Simple- Interactive, computer-assisted instruction	Pamphlet	Unclear	Free mammography. Assume pamphlet was control group.
Champion, 2007 [52]	Clinic and health maintenance organisations	No mammogram in the previous 18 months.	Simple- Tailored phone call Simple- Tailored print media Multiple- Tailored phone and print	Usual care	1996-2002	
Dietrich, 2006 [35]	Community and migrant health centres	Overdue for at least one screening	Multiple- Four phone calls and written material	Usual care and one phone call and written material	2001-2002	
Dietrich, 2007 [53]	Medicaid managed care organization (MMCO)	Overdue for breast, cervical or colorectal screening	Simple- Three scripted telephone calls to identify barriers and provide support.	Modified version of telephone outreach programme, also in up to 3 calls.	May to December 2005	
Jibaja-Weiss, 2003 [32]	Community Health Centres	No mammogram or PAP in the previous 2 years.	Simple- Personalised tailored letter; Simple- Personalised form letter	No communication	Unclear	
Kim, 2004 [37]	Korean Churches in Los Angeles	No mammogram in the previous 12 months.	Simple- Free/low cost mammography Multiple- Peer group education and free/low cost mammography	Cholesterol education, blood tests and osteoporosis screening	Unclear	Low cost/free mammography at church
Kreuter, 2005 [28]	Urban public health centres	Not inclusion criteria but 54.6% had a mammogram in the last 12 months	Simple- 6 behaviourally tailored magazines (BCT); Simple- 6 culturally relevant magazines (CRT); Multiple- BCT and CRT magazines	Usual care	1998-2000	
Maxwell, 2003 [34]	Nine community based organisations and six churches	Not inclusion criteria but 48% had a mammogram in the last 12 months	Face to face- Cancer screening education session with Filipino health educator, physicians and nurses	Physical activity education session	Unclear	Information packages provided where free mammograms were available.
Mishra, 2007 [54]	Samoan speaking churches	No mammogram in the previous 2 years.	Face to face- 4 weekly group sessions on mammography run by Samoan nurses	Usual care	1998-2001	
Nuño, 2011 [33]	Community survey	Not stated	Face to face- Two hour group education session with community lay health worker	Usual care and mail and phone reminder to have mammography	2002-2005	
Oleske, 2007 [55]	Hospitals	Not inclusion criteria	Face to face- Breast cancer survivors trained to educate relatives	Pamphlets for relatives	2000-2002	
Paskett, 2006 [36]	Consortium of community health centres	No mammogram in the previous 12 months	Face to face- Individual health education program that was tailored to the needs of each woman	Written material on cervical screening and advice	1998-2002	
Phillips, 2010 [56]	Three internal medicine practices	No mammogram in the last 18 months	Multiple- Patient navigator system including phone call and letter	Usual care	February to November 2008	Free for publically and uninsured.
Powell, 2005 [29]	13 African American churches	Not in inclusion criteria	Face to face- Group education Multiple- Group education and home visit by a home health educator.	Group information session	Unclear	
Puschel, 2010 [24]	Community clinic	No mammogram in the last 2 years	Simple- mail contact; Multiple- mail plus phone contact plus home visit	Usual care plus opportunistic care- mammogram advice at clinic	From 2008	Received free health care
Russell, 2010 [31]	Health Centre	No mammogram in the last 15 months	Multiple- Tailored computer programme and four lay health advisor counselling sessions	Culturally appropriate pamphlet and postcard with nutritional information	2006-200	
Slater, 2005 [23]	Community based	Not in inclusion criteria	Simple- Two simple mailings to access free mammogram Multiple- Two simple mailings to access free mammogram plus a $10 incentive for those who completed a mammogram within 1 year	Not stated	1999-2001	Free mammograms for all with Sage
West, 2004 [27]	Family Health Centre	No mammogram in the previous 2 years	Simple- At stage 1, a personalised letter; Simple- At stage 2, a tailored phone call	At stage 1, usual care; At stage 2, a tailored letter	October 1997 and May 1999	No cost for mammography
Young, 2002 [25]	Primary care	No mammography in the previous 12 months	Multiple- Cancer education programme and appointment for free on-site mammography	Observational assessment which included a telephone questionnaire	Not stated.	
Zhu, 2002 [26]	Public housing complexes	Not stated	Face to face- Lay health education home visits by African American women	Not stated	1997-	

**Table 2:** Description of studies included in the review.

Reference	Number of participants	Age (Years)	Location	Ethnicity	Level of randomisation	Length of follow-up (Months)	Source of outcome
Ahmed, 2010	2357	40+	Not stated	43% African American 12% Hispanic; 45% White	Individual	12	Medical Records
Champion, 2006	344 (492 invited) Response rate 69.9%	41–75	Urban	African American	Individual	6	Self-reported
Champion, 2007	1245	66 (mean)	Urban	54% African American 44% White	Individual	4	Medical Records
Dietrich, 2006	1413	50–69	Urban	Not stated; 63% primary language Spanish	Individual	18	Medical Records
Dietrich, 2007	1316	40–69	Urban	Not stated; 15% primary language Spanish	Individual	8	Medical Records
Jibaja-Weiss, 2003	739	40–64	Urban	41% African American 42% Mexican American 18% Non-Hispanic White	Individual	12	Medical Records
Kim, 2004	141	40–65	Urban	Korean	Church	2	Self-reported
Kreuter, 2005	416 (Analyses based on 192)	40–65	Urban	African American	Individual	6 or 18	Self-reported
Maxwell, 2003	447 (530 invited) Response rate 84%	40+	Urban	Filipino American	Small groups of women	12	Self-reported
Mishra, 2007	776 (68 churches)	42+	Not stated	Samoan	Church	8	Self-reported
Nuño, 2011	381 (out of 446 eligible); Response rate 85.4%	50+	Rural	Hispanic	Individual	Unclear- outcome data for previous 12 months	Self-reported
Oleske, 2007	96	21+	Urban	34% African American 48% Hispanic; 16% White	Individual	3-6	Self-reported
Paskett, 2006	897	40+	Rural	33% African American 42% Native American; 25% White	Individual	12-14	Medical Records
Phillips, 2010	3895	51–70	Urban	47% African American 11% Hispanic; 29% White	Primary care provider	9	Medical Records
Powell, 2005	197 (13 churches)	40+	Rural	African American	Church	3	Self-reported
Puschel, 2010	500 (540 invited) Response rate 92.6%	50–70	Urban Chile	9% Indigenous; 46% Mestizo; 46% White	Individual	6	Medical Records
Russell, 2010	181 (251 eligible) Response rate 72.1%	41–75	Urban	African American	Individual	6	Self-reported; Medical records verified
Slater, 2005	145,467	40–63	Mixed	Not stated	Individual	12	Medical Records and self-reported
West, 2004	320 stage 1; 237 stage 2	50–80	Rural	91% African American	Individual	6 for each stage	Self-reported
Young, 2002	94	40+	Urban	94% African American	Individual	3	Self reported Validated by records of mobile van
Zhu, 2002	325 (10 Housing Complexes); Follow-up data for 255 (367 eligible)	65+	Urban	African American	Public Housing complexes	12 and 24	Self-reported

### Statistical analysis

Due to the expected heterogeneity in the effect of interventions, we performed random effects meta-analyses [18]. We investigated between study heterogeneity using I^2^ statistics [19]; values of <25%, 25–75% and >75% represent low, medium and high heterogeneity respectively. We examined potential sources of heterogeneity, including intervention type (simple, face to face or multiple), length of follow-up, outcome source (self-reported or medical records), study quality, time lag and location (urban or rural) by sub-group analyses and meta-regression [20], for which we used post-estimation Wald tests to obtain F ratios and p values.

Study quality was assessed independently by two reviewers using the Cochrane risk of bias tool [21]; discrepancies were resolved by consensus. Our assessment was based on four of the six criteria: random sequence generation; allocation concealment; blinding of trial personnel or outcome assessors and incomplete outcome data. Blinding of subjects was not assessed, as this would not be feasible in studies of behavioural interventions. Selective outcome reporting was not assessed since the majority of studies would likely fall into the unclear category due to the lack of availability of study protocols. Other potential sources of bias on each study were also identified. For each criterion, studies were assessed as being at high, low or unclear risk of bias. We included the three most important domains (random sequence generation, allocation concealment and incomplete outcome data) as covariates in the meta-regression. We used funnel plots to assess publication bias and tested the symmetry of the funnel plots using Egger's test [21], [22].

We undertook a series of sensitivity analyses. We repeated the analyses having omitted one very large study (two comparisons) [23] and the one non-US based study [24]. We also repeated the analyses having omitted the studies with a high risk of bias for at least one of the key quality domains (*a priori*).

## Results

**Figure 1:**
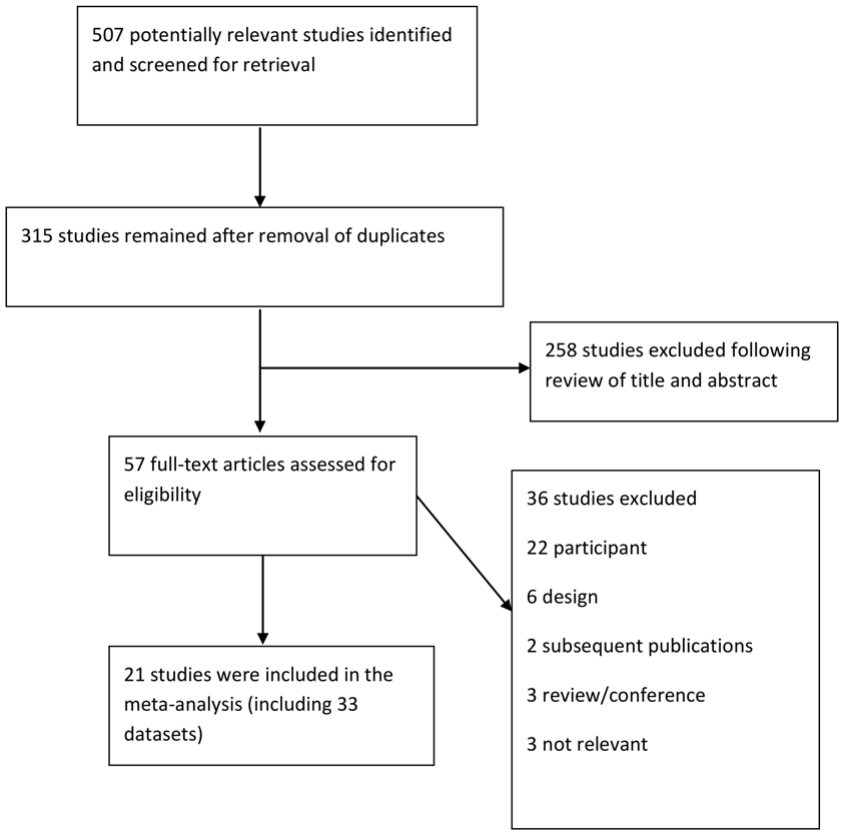
Flow diagram for identification of published studies for inclusion in review.

The search identified 315 studies. Two hundred and fifty eight studies were excluded from their title and abstract, as they were not relevant to the review. Following assessment of full text articles, 36 were excluded (see Figure 1 for details) and 21 studies (33 comparisons) were included in our meta-analyses.

The characteristics of the included studies are detailed in Tables 1 and 2. In seven studies, ethnicity was used as an inclusion criteria, all were solely African-American women [25]–[31]. Ethnicity was reported in a further 11 studies, see Table 2.

The post-intervention risk differences (expressed as a percentage) between intervention and control ranged from −8% (95% CI −14% to −1%; Table 3) [32], i.e. fewer women in the intervention received mammography compared to the control group, where the intervention was a personalised tailored letter, to 64% (95% CI 56% to 72%; Table 3) [24] where the intervention was a multi-component strategy including mail, phone call and a home visit. This most effective intervention was the study from Chile [24] and the treatment received by the control group was a combination of usual and opportunistic care. In the study where the control was more effective than the intervention, the treatment received by the control group was a pamphlet [32].

**Table 3:** Results of studies included in the review.

Reference	Intervention Had mammogram (n)	Intervention No mammogram (n)	Control Had mammogram (n)	Control No mammogram (n)	Risk difference, *p*1 –*p*_0_ (95% CI)
Ahmed, 2010 (Letter)	126	659	105	681	0.03 (−0.01, 0.06)
Ahmed, 2010 (Multiple)	213	573	105	681	0.14 (0.10, 0.18)
Champion, 2006 (Video)	29	89	18	38	−0.08 (−0.22, 0.07)
Champion, 2006 (Computer)	50	75	18	38	0.08 (−0.07, 0.23)
Champion, 2007 (Phone)	91	223	68	226	0.06 (−0.01, 0.13)
Champion, 2007 (Print)	105	224	68	226	0.09 (0.02, 0.16)
Champion, 2007 (Multiple)	108	200	68	226	0.12 (0.05, 0.19)
Dietrich, 2006 (Multiple)	473	223	403	291	0.10 (0.05, 0.15)
Dietrich, 2007 (Phone)	343	320	326	327	0.02 (−0.04, 0.07)
Jibaja-Weiss, 2003 (Letter)	31	208	54	207	−0.08 (−0.14, −0.01)
Jibaja-Weiss, 2003 (Form)	73	166	54	207	0.10 (0.02, 0.17)
Kim, 2004 (Multiple)	41	6	22	24	0.39 (0.22, 0.57)
Kim, 2004 (Access*)	35	13	22	24	0.25 (0.06, 0.44)
Kreuter, 2005 (BCT magazine)	31	17	30	25	0.10 (−0.09, 0.29)
Kreuter, 2005 (CRT magazine)	28	16	30	25	0.09 (−0.10, 0.28)
Kreuter, 2005 (Multiple)	34	11	30	25	0.21 (0.03, 0.39)
Maxwell, 2003 (Face to face)	126	87	134	100	0.02 (−0.07, 0.11)
Mishra, 2007 (Face to face)	185	206	148	236	0.09 (0.02, 0.16)
Nuño, 2011 (Face to face)	134	49	109	79	0.15 (0.06, 0.25)
Oleske, 2007 (Face to face)	29	16	36	16	−0.05 (−0.24, 0.14)
Paskett, 2006 (Face to face)	184	249	114	304	0.15 (0.09, 0.22)
Phillips, 2010 (Multiple)	1575	242	1589	489	0.10 (0.08, 0.13)
Powell, 2005 (Face to face)	50	21	27	17	0.09 (−0.09, 0.27)
Powell, 2005 (Multiple)	47	28	27	17	0.01 (−0.17, 0.19)
Puschel, 2010 (Mail)	86	80	10	157	0.46 (0.37, 0.54)
Puschel, 2010 (Multiple)	117	50	10	157	0.64 (0.56, 0.72)
Russell, 2010 (Multiple)	45	44	16	74	0.33 (0.20, 0.46)
Slater, 2005 (Mail)	342	25291	661	93540	0.01 (0.00, 0.01)
Slater, 2005 (Multiple)	488	25145	661	93540	0.01 (0.01, 0.01)
West, 2004 (Letter)	22	137	23	138	−0.00 (−0.08, 0.07)
West, 2004 (Phone)	18	101	15	103	0.02 (−0.06, 0.11)
Young, 2002 (Multiple)	31	16	18	29	0.28 (0.08, 0.47)
Zhu, 2002 (Face to face)	107	55	111	52	−0.02 (−0.12, 0.08)

* Access to free or low cost mammography.

**Figure 2:**
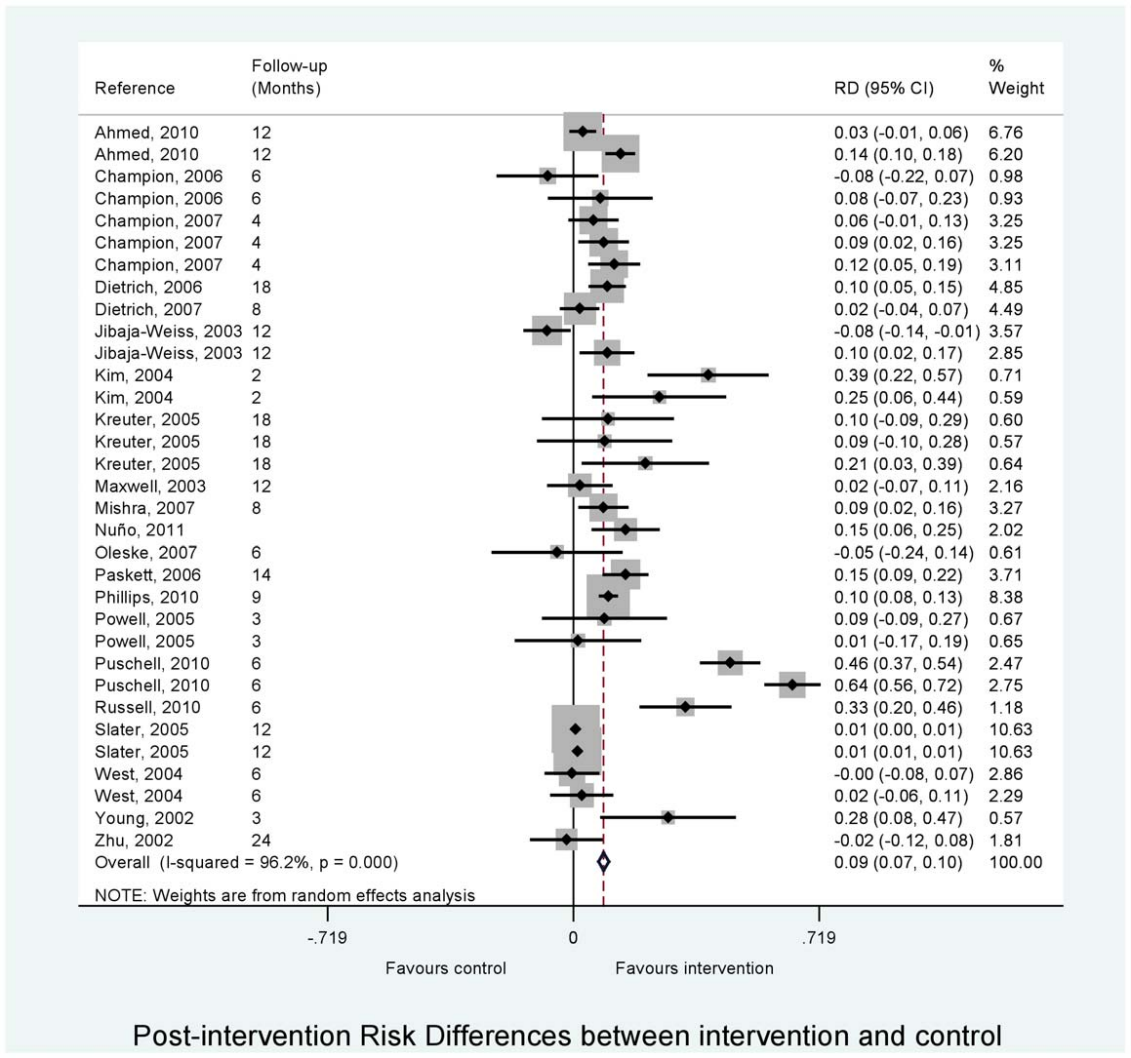
Meta-analysis of the association between intervention and mammography uptake in low income women.

The overall pooled analysis showed that interventions increased the uptake of mammography in low-income women by a difference of 8.9% (CI 7.3 to 10.4%) compared with women in the control group (Table 4; Figure 2). There was evidence of substantial heterogeneity in meta-analysis of association between intervention and mammography uptake (I^2^  = 96.2%, p<0.001).

**Table 4:** Results from overall and stratified random effects meta-analyses for the associations between intervention and mammography uptake in low income women.

Stratification	No*	RD^†^	95% CI	*P*-value	I^2^	*P-*value^‡^
**Overall effect**	33	0.089	0.073 to 0.104	<0.001	96.2%	<0.001
**Intervention^a^**						
Simple	15	0.069	0.018 to 0.119	0.01	91.4%	<0.001
Face to face	7	0.075	0.017 to 0.132	0.01	57.9%	0.03
Multiple	11	0.207	0.113 to 0.300	<0.001	98.2%	<0.001
Overall					**F ratio** 3.00	***P-value*** 0.06
**Follow-up (months)**						
≤6	16	0.170	0.058 to 0.281	0.003	94.4%	<0.001
>6	17	0.044	0.031 to 0.056	<0.001	94.3%	<0.001
Overall					**F ratio 3.99**	*P-value* 0.05
**Source outcome**						
Medical records	14	0.156	0.085 to 0.227	<0.001	96.0%	<0.001
Self-reported	16	0.073	0.024 to 0.123	0.004	59.5%	0.001
Overall					**F ratio **1.73	***P-value*** **0.20
**Location**						
Urban	22	0.144	0.075 to 0.213	<0.001	93.8%	<0.001
Rural	6	0.089	0.073 to 0.104	0.03	66.3%	0.01
Overall					**F ratio** 0.82	***P-value*** 0.37

*33 comparisons from 21 studies. † Effect measure is the difference in proportions between intervention and control group. ‡ *P*-value is obtained from the heterogeneity χ^2^. ^a^ Simple interventions include letters, telephone calls, videos and computer programmes but these are not face to face interventions. Multiple interventions include more than one type of intervention.

**Figure 3:**
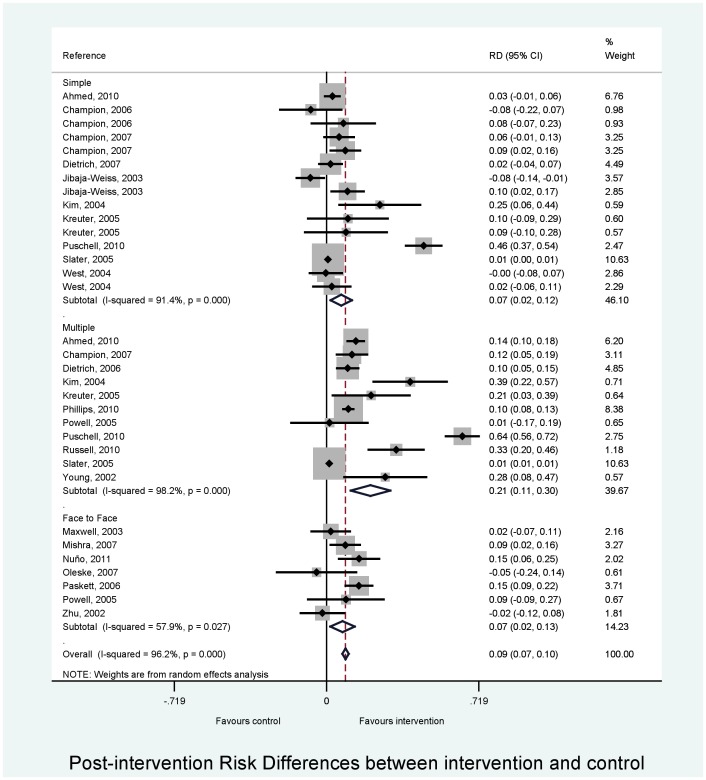
Stratified meta-analysis by type of intervention for the association between intervention and mammography uptake in low income women.

Sub-group analyses are shown in Table 4. There was weak evidence that that the association between intervention and mammography uptake varied by intervention type (F ratio  = 3.00, p = 0.06; Table 4; Figure 3). The association was stronger for multi-component compared with simple interventions. This effect was driven by the largest effect of intervention in the multiple group (F = 5.06, p = 0.03; comparing multiple to simple intervention). There was weak evidence to suggest that the effect of the intervention was stronger in studies with a shorter length of follow-up (≤6 months) (Figure s1A in File S1). There was little evidence that that the association between intervention and mammography uptake varied by source outcome (medical records or self-reported) (Figure s1B in File S1) or by location (urban or rural) (Figure s1C in File S1). When intervention type, follow-up group and source outcome were included as covariates in a meta-regression analysis, heterogeneity remained substantial (I^2^  = 89.2%). We found little effect on pooled risk difference whether mammography was free or not (Figure s1D in File S1), whether control was usual care or not (Figure s1E in File S1) or by level of randomisation (Figure s1 F in File S1).

### Risk of bias

The outcome of the risk of bias assessment is shown in Table 5. Two studies were assessed as having a high risk of bias in the random sequence generation [29], [33]. Four studies were considered to have a high risk of bias for allocation concealment [31], [33]–[35] and one further study had a high risk of bias in the second stage of their two-stage study design [27]. The risk of bias from not blinding trial personnel or outcome assessors was low/unclear in all studies. Four studies were assessed has having a high risk of bias for addressing incomplete outcome data [25], [28], [32], [36], three of those [28], [32], [36] did not use intention to treat analysis and in the other study there was unequal loss to follow-up [25]. Using meta-regression, there was little evidence that the association between intervention and mammography uptake varied by risk of bias classification (low, unclear or high) for random sequence generation (F ratio  = 0.12, p = 0.89), allocation concealment (F ratio  = 1.36, p = 0.27) or incomplete outcome data (F ratio  = 1.22, p = 0.31).

**Table 5:** Risk of bias* assessment.

Reference	Sequence generation (Randomisation)†	Allocation concealment‡	Blinding of trial personnel or outcome assessors Ω	Incomplete outcome data andintention to treat ††	Other sources of bias €
Ahmed, 2010	Low	Low	Unclear	Low	Highest dropout in the intervention group, but ITT analysis used. Possible contamination in all groups, so effect may be diluted.
Champion, 2006	Unclear	Unclear	Unclear	Unclear	Possible differential completeness of outcome assessment as self-reported mammograms.
Champion, 2007	Unclear	Unclear	Unclear	Unclear	
Dietrich, 2006	Low	High	Low	Low; ITT analysis.	
Dietrich, 2007	Unclear	Unclear	Unclear	Low	High dropout similar across groups. ITT analysis used. Control group received substantial intervention.
Jibaja-Weiss, 2003	Low	Unclear	Low	High. Did not use ITT analysis.	The two interventions were similar in content.
Kim, 2004	Unclear	Unclear	Unclear	Unclear	Possible differential completeness of outcome assessment as self-reported mammograms.
Kreuter, 2005	Low	Low	Unclear	High. Did not use ITT analysis.	Possible differential completeness of outcome assessment as self-reported mammograms. Error in computer programme meant 37 women were given the wrong follow-up survey and hence were excluded. 16 women were excluded as they had a mammography after 1 month (not due to intervention). Attrition was non-differential by study group.
Maxwell, 2003	Unclear	High	Low	Unclear. ITT analysis.	Possible differential completeness of outcome assessment as self-reported mammograms.
Mishra, 2007	Unclear	Unclear	Low	Unclear. ITT not used.	Possible differential completeness of outcome assessment as self-reported mammograms.
Nuño, 2011	High	High	Unclear	Low; ITT analysis	Potential differential completeness of outcome assessment as self-reported mammograms. Although 65% available through medical records.
Oleske, 2007	Unclear	Unclear	Unclear	Unclear	Potential differential completeness of outcome assessment as self-reported mammograms.
Paskett, 2006	Unclear	Unclear	Low	High. Did not use ITT analysis.	Unequal loss to follow-up.
Phillips, 2010	Unclear	Unclear	Unclear	Low	
Powell, 2005	High- unbalanced groups at baseline	Unclear	Unclear	Unclear	Results were not adjusted for baseline differences. Possible differential completeness of outcome assessment as self-reported mammograms.
Puschel, 2010	Low	Low	Unclear	Low; ITT analysis	Balanced loss to follow-up.
Russell, 2010	Low	High	Low	Low; ITT analysis	
Slater, 2005	Low	Unclear	Unclear	Unclear	Potential contamination of control group- Sage recruitment activities including Community Health agency recruiters, print and broadcast media advertisements and individual participating clinics promoting members newsletters and the Sage program.
West, 2004	Unclear	Unclear for stage 1 but high for stage 2	Unclear	Unclear. ITT analysis	Possible differential completeness of outcome assessment as self-reported mammograms; Potential contamination of control group- ADPH-sponsored Breast and Cervical Cancer Screening Program was under way in rural Alabama and being widely promoted.
Young, 2002	Unclear	Unclear	Unclear	High	Unequal loss to follow-up.
Zhu, 2002	Unclear	Unclear	Unclear	Unclear. Did not use ITT analysis.	Possible differential completeness of outcome assessment as self-reported mammograms. Accounted for clustering by housing complex in analysis.

* Low for low risk of bias, high for high risk of bias and unclear for risk unclear.

† Assessment of whether method used to generate the allocation sequence should produce comparable groups.

‡ Assessment of whether allocation could have been foreseen in advance of enrolment by participants or recruitment personnel.

Ω Assessment of whether knowledge of the allocated intervention was adequately prevented during the study.

†† Assessment of whether incomplete outcome data were adequately dealt with, including assessment of attrition rates in included studies.

€ State any important concerns about bias not addressed in the other domains in the tool.

### Publication bias

The Egger test showed evidence of small study bias (bias  = 2.61, p = 0.001) but this test was skewed by the two comparisons from a very large study [23]. Having omitted these, there was little evidence for a small study bias with the funnel plot (Figure s2A in File S1) or Egger plot (Figure s2B in File S1; bias  = 0.96, p = 0.43).

### Time lag bias

We recorded the time lag between the end of the intervention period and the date of publication and this was classified into ≤4 years and >4 years. For seven studies this could not be determined. There was weak evidence that a shorter time lag was associated with a stronger association between intervention and mammography uptake (F ratio = 2.94, p = 0.10; Figure s2C in File S1), indicating that studies showing a strong effect tend to be published sooner.

### Sensitivity Analyses

Omitting one large study [23] had little effect on the associations between intervention and mammography uptake (data not shown). Apart from one [24] where participants were from primary care in Chile, all the trials were conducted in the US. Having omitted that, the overall pooled analysis showed that interventions increased the uptake of mammography in low-income women by a difference of 5.6% (CI 4.3 to 6.0%) compared with women in the control group. There was now stronger evidence that the association between intervention and mammography uptake varied by intervention type (F ratio  = 4.07, p = 0.03). Again, the association was stronger for multi-component compared with simple interventions (F ratio  = 8.13, p = 0.01). Including intervention type, follow-up group and source outcome as covariates in a meta-regression analysis, the heterogeneity was now reduced to a moderate level (I^2^  = 61.2%). There was little effect on the other analyses (data not shown). Omitting the studies with a high risk of bias for either random sequence generation or allocation concealment or incomplete outcome data had little effect on the analyses (data not shown).

## Discussion

The results of these meta-analyses showed that interventions can increase the uptake of mammography in low-income women. There was weak evidence that this association varied by type of intervention. This effect was driven by the largest effect of intervention in the multiple group (comparing multiple to simple intervention). Indeed, ‘face to face’ interventions were no more effective than simple interventions in increasing mammography uptake in low-income women. There was weak evidence to suggest that the associations were stronger in studies with a shorter length of follow-up. There was little evidence that the association between intervention and mammography uptake varied by source outcome (medical records or self-reported), location (urban or rural), whether mammography was free or not, whether control was usual care or not or by level of randomisation.

### Explanation of findings

Multiple interventions showed the largest difference between intervention and control groups (20.7%). This is consistent with the findings of Legler [15], where multiple interventions were also reported as the most effective strategy, leading to a 27% increase in uptake of mammography. In their review [15], multiple interventions included access-enhancing interventions (e.g. free onsite mammography) which alone led to a 19% increase in mammography uptake. In our review, only one study [37] trialled an access-enhancing intervention alone (free onsite mammography) and was classified as a simple intervention. In the review by Bailey [16], peer educators (‘face to face’), access-enhancing and multiple interventions were effective in increasing screenings. However, since they did not undertake a meta-analysis [16], they could not determine which was the most effective strategy.

In our study, the most effective multi-strategy study reported a 64% difference between the control and intervention group and included a mail, phone call and home visit [24]. The home visit was undertaken if a phone was not available or if the participants had not made an appointment for a mammogram within an extra four weeks from the previous contact. The more effective multi-strategy studies tended to include ‘face to face’ interventions (e.g. [24], [31]), although not always (e.g. [28]). One study [28] combined two simple interventions to produce six magazines that contained both generic breast cancer knowledge and culturally tailored information and reported a 21% difference between the control and intervention group. Furthermore, effective multi-strategy interventions gave similar differences between the control and intervention group whether the ‘face to face’ components were intensive (33%; four lay health adviser sessions; [31]) or not (39%; a one hour session; [37]). This has implications for the cost-effectiveness of the interventions [38], [39], home visits being a costly strategy [40]. More studies are needed to determine which interventions are most cost-effective [15]. One meta-analysis on mammography interventions (albeit not restricted to low-income women) which undertook a cost-effectiveness analysis [39] found that when a phone number was available, a letter plus telephone call intervention was more cost-effective than a two letter intervention.

Given that a recent review found good evidence to support the continuation of population-based mammography screening in the UK [41], it is vital that this service is equally accessible to all women, irrespective of income, ethnicity and socio-economic position. A systematic review and meta-analysis of interventions to increase the uptake of mammography in population based screening programs was undertaken by The Cochrane Collaboration [40]. They found that effective strategies for increasing uptake of mammography were letters of invitation, a phone call or a combination of a letter and phone call. Home visits interventions were no more effective than the control group treatment. The findings of this review are based on studies of low-income women. In our review, multiple interventions were the most effective strategy but simple interventions (letter or phone call) or ‘face to face’ interventions were more effective than the control group treatment. Interventions based on such strategies are required to reduce inequalities in uptake of mammography screening in the UK.

Apart from the study from Chile [24], all studies were carried out in the US, limiting the generalisability of the results to other settings. The US healthcare system is based on private insurance, supplemented by Medicare for over 65 s. In contrast, healthcare in the UK remains free at the point of service. It is therefore likely that cost is more of a barrier for low-income women to attend mammography in the US compared to other countries. Nevertheless, opportunity costs are important to consider, even in health care settings such as the UK's National Health Service. Women who are paid a wage (e.g. by the hour) incur a higher personal cost through loss of income than salaried women.

We were unable to test empirically whether similar effect sizes would be found in countries with free mammography. The largest effect sizes in the current study were from the only non-US study, based in Chile [24], where free mammography has been provided since 2005. Further research is required in countries other than in the US. There is limited evidence from other countries with population-based screening programs regarding interventions which improve mammography uptake. For example, in the UK, training activities plus reminder letters or phone calls increased the uptake of mammography in one population-based study by an additional 5% [42] but in another such study using a ‘face to face’ intervention, results were consistent with chance [43]. In Australia, where mammography screening is free for women aged 50-69 years, a population based RCT found that a multi-component strategy of letter plus phone increased the uptake of mammography by an additional 6% [39]. However, it must be remembered that these total-population approaches do not necessarily improve uptake equally and can indeed increase inequalities while improving uptake overall. A ‘face to face’ intervention was not an effective strategy for promoting uptake in mammography by Asian women in the UK [44]. A letter from the general practitioner did not improve uptake of mammography in an area of high deprivation in the UK [45].

A meta-analysis of interventions to promote mammography among ethnic minority women in the US, found that access-enhancing interventions were the most effective [46]. In the majority of studies in our review, a significant proportion of women who participated were of African-American ethnicity. Hence the results might not be immediately applicable to women from other ethnic minorities in the US. However, in the study to increase mammography use by Korean American women [37], the multi-strategy intervention reported a 39% difference between the control and intervention group. Successful interventions are likely to be context-specific, hence ‘face to face’ interventions were effective in Hispanic women along the U.S.-Mexico border (15%) [33] but such interventions were less effective in Filipino American women (2%) [34]. Nevertheless, the data presented in this review can be drawn on for the development of suitable interventions in settings other than those described here.

There was little evidence that the association between intervention and mammography uptake varied by risk of bias classification. We also undertook sensitivity analysis and omitted those studies with a high risk of bias for either random sequence generation, or allocation concealment or incomplete outcome data domains. This had little effect on the associations, suggesting that the reliability of the results is not unduly affected by the quality of the included studies.

### Heterogeneity

Due to the expected heterogeneity in the effects of interventions, we performed random effects meta-analyses chosen *a priori*. We examined potential sources of heterogeneity, an important component of carrying out a meta-analysis [47]. The variables investigated were type of intervention, length of follow-up, location and source outcome (medical records versus self-reported). Women tend to over-report their participation in mammography screening [48]. In addition, we examined potential sources for heterogeneity whether the control was usual care or not, whether mammography was free or not and by the level of randomisation. Whilst there was weak evidence that the association between intervention and mammography uptake varied by type of intervention, there was little evidence that the association varied by other covariates. Several of the estimates of I^2^ calculated in meta-analyses are considered as moderate (e.g. ‘face to face’ I^2^  = 57.9%) to high (e.g. simple I^2^  = 91.4%). In addition to the characteristics investigated, it is possible that other factors might vary by study context, for example ethnic diversity, type of ‘face to face’ intervention and whether the letter was system-directed or individual directed [49] and could result in heterogeneity. Another option for investigating heterogeneity is the quality effects model [50]. For this a quality score is required. However, the use of scales for assessing quality is discouraged in Cochrane Reviews since it involves assigning weights to different items in the scale and it is difficult to justify the weights assigned [21]. Indeed, the influence of quality is best done using sensitivity analyses [47] as we did in the current study for example by omitting studies with a high risk of bias.

### Strength and Limitations

This large scale systematic review and meta-analysis looking into the effectiveness of interventions used to increase uptake of mammography amongst low-income women includes data from 21 published studies and including 33 datasets. We have tested a priori hypotheses and have assessed the quality of each included study. We have thus aimed to minimise a range of biases including selection bias. We also undertook a series of sensitivity analyses, including omitting those studies with a high risk of bias for random sequence generation, allocation concealment or incomplete outcome data. Since we searched only for published articles, there is the possibility of publication bias. Although this was a large systematic review and meta-analysis, we might still have been underpowered for the sub-group analyses, meta-regression and publication bias. Hence whilst the funnel plots and formal tests for publication bias gave no strong evidence for publication bias, these need to be interpreted with caution. All of the studies included in the review had a short length of follow-up. It is unclear whether our findings would be maintained over the medium term. This is supported by the suggestion from our data that the associations were stronger in studies with a shorter length of follow-up. Furthermore, apart from one study, all of the studies were conducted in the US and hence the applicability of these results to other countries may be limited.

In conclusion this paper found multiple interventions were the most effective strategy in increasing uptake of mammography in low-income women. The limited evidence from this review does not allow clear recommendations for practice to be made. The cost-effectiveness of interventions needs to be estimated before recommending which interventions could be used to reduce inequalities in mammography uptake. Further research is required in countries other than the US.

## Supporting Information

File S1 **Stratified meta-analyses examining potential sources of heterogeneity for length of follow-up (Figure s1A), source outcome (Figure s1B), location (Figure s1C), whether mammography was free or not (Figure s1D), whether control was usual care or not (Figure s1E) and by level of randomisation (Figure s1F).** Testing for small study bias by Funnel plot (Figure s2A) and Egger plot (Figure s2B) and assessing publication bias by stratified meta-analysis (Figure s2C).
(DOCX)

Table S1 **Search strategy used to search Medline and Embase databases, to April 2012.**
(DOCX)

## References

[1] Kamangar F, Dores GM, Anderson WF (2006) Patterns of cancer incidence, mortality, and prevalence across five continents: Defining priorities to reduce cancer disparities in different geographic regions of the world. J Clin Oncol 24: 2137–50. 16682732 10.1200/JCO.2005.05.2308

[2] Elmore JG, Armstrong K, Lehman CD, Fletcher SW (2005) Screening for breast cancer. JAMA 293: 1245–56. 15755947 10.1001/jama.293.10.1245 PMC3149836

[3] Gotzsche PC, Nielsen M (2011) Screening for breast cancer with mammography. Cochrane Database Syst Rev 1: CD001877. 10.1002/14651858.CD001877.pub4 21249649

[4] Sant M, Allemani C, Santaquilani M, Knijn A, Marchesi F, et al (2009) EUROCARE-4. Survival of cancer patients diagnosed in 1995-1999. Results and commentary. Eur J Cancer 45: 931–91. 19171476 10.1016/j.ejca.2008.11.018

[5] Sant M, Allemani C, Capocaccia R, Hakulinen T, Aareleid T, et al (2003) Stage at diagnosis is a key explanation of differences in breast cancer survival across Europe. Int J Cancer 106: 416–22. 12845683 10.1002/ijc.11226

[6] Gotzsche PC, Jorgensen KJ, Zahl PH, Maehlen J (2012) Why mammography screening has not lived up to expectations from the randomised trials. Cancer Causes Control 23: 15–21. 22072221 10.1007/s10552-011-9867-8

[7] McKenzie F, Ives A, Jeffreys M (2012) Socio-economic inequalities in survival from screen-detected breast cancer in South West England: population-based cohort study. Eur J Public Health 22: 418–22. 21891789 10.1093/eurpub/ckr107

[8] The Information Centre. Breast Screening Programme. Available: http://www.ic.nhs.uk/webfiles/publications/008_Screening/brstscreen1011/brst_scr_prog_eng_2010_11_rep.pdf. Accessed 2012 January 8.

[9] Botha JL, Manku-Scott TK, Moledina F, Williams A (1993) Indirect discrimination and breast screening. Ethn Dis 3: 189–95. 8324497

[10] Swan J, Breen N, Coates RJ, Rimer BK, Lee NC (2003) Progress in cancer screening practices in the United States: results from the 2000 National Health Interview Survey. Cancer 97: 1528–40. 12627518 10.1002/cncr.11208

[11] Reuben DB, Bassett LW, Hirsch SH, Jackson CA, Bastani R (2002) A randomized clinical trial to assess the benefit of offering on-site mobile mammography in addition to health education for older women. Am J Roentgenol 179: 1509–14. 12438046 10.2214/ajr.179.6.1791509

[12] Katz SJ, Zemencuk JK, Hofer TP (2000) Breast cancer screening in the United States and Canada, 1994: socioeconomic gradients persist. Am J Public Health 90: 799–803. 10800435 10.2105/ajph.90.5.799 PMC1446215

[13] Sin JP, St Leger AS (1999) Interventions to increase breast screening uptake: do they make any difference? J Med Screen 6: 170–81. 10693060 10.1136/jms.6.4.170

[14] Lyratzopoulos G, Barbiere JM, Rachet B, Baum M, Thompson MR, et al (2011) Changes over time in socioeconomic inequalities in breast and rectal cancer survival in England and Wales during a 32-year period (1973 to 2004): the potential role of health care. Ann Oncol 22: 1661–6. 21199888 10.1093/annonc/mdq647

[15] Legler J, Meissner HI, Coyne C, Breen N, Chollette V, et al (2002) The effectiveness of interventions to promote mammography among women with historically lower rates of screening. Cancer Epidemiol Biomarker Prev 11: 59–71. 11815402

[16] Bailey TM, Delva J, Gretebeck K, Siefert K, Ismail A (2005) A systematic review of mammography educational interventions for low-income women. Am J Health Prom 20: 96–107. 10.4278/0890-1171-20.2.96 PMC1820866 16295701

[17] Moher D, Liberati A, Tetzlaff J, Altman DG (2009) The PRISMA Group (2009) Preferred Reporting Items for Systematic Reviews and Meta-Analyses: The PRISMA Statement. PLoS Med 6: e1000097. 19621072 10.1371/journal.pmed.1000097 PMC2707599

[18] DerSimonian R, Laird N (1986) Meta-analysis in clinical trials. Controlled Clinical Trials 7: 177–88. 3802833 10.1016/0197-2456(86)90046-2

[19] Higgins JP, Thompson SG, Deeks JJ, Altman DG (2003) Measuring inconsistency in meta-analyses. BMJ 327: 557–60. 12958120 10.1136/bmj.327.7414.557 PMC192859

[20] Thompson SG, Sharp SJ (1999) Explaining heterogeneity in meta-analysis: a comparison of methods. Stat Med 18: 2693–708. 10521860 10.1002/(sici)1097-0258(19991030)18:20<2693::aid-sim235>3.0.co;2-v

[21] Higgins JPT, Altman DG (eds) (2008) Chapter 8: Assessing risk of bias in included studies. In: Higgins JPT, Green S (editors). Cochrane Handbook for Systematic Reviews of Interventions.Version 5.0.1 [updated September 2008]. The Cochrane Collaboration. Available from www.cochrane-handbook.org.

[22] Egger M, Davey Smith G, Schneider M, Minder C (1997) Bias in meta-analysis detected by a simple, graphical test. BMJ 315: 629. 9310563 10.1136/bmj.315.7109.629 PMC2127453

[23] Slater JS, Henly GA, Ha CN, Malone ME, Nyman JA, et al (2005) Effect of direct mail as a population-based strategy to increase mammography use among low-income underinsured women ages 40 to 64 years. Cancer Epidemiol Biomarker Prev 14: 2346–52. 10.1158/1055-9965.EPI-05-0034 16214915

[24] Puschel K, Coronado G, Soto G, Gonzalez K, Martinez J, et al (2010) Strategies for increasing mammography screening in primary care in Chile: results of a randomized clinical trial. Cancer Epidemiol Biomarker Prev 19: 2254–2261. 10.1158/1055-9965.EPI-10-0313 PMC3985495 20826832

[25] Young RF, Waller JB Jr, Smitherman H (2002) A breast cancer education and on-site screening intervention for unscreened African American women. J Cancer Educ 17: 231–6. 12556062 10.1080/08858190209528844

[26] Zhu K, Hunter S, Bernard LJ, Payne-Wilks K, Roland CL, et al (2002) An intervention study on screening for breast cancer among single African-American women aged 65 and older. Prev Med 34: 536–45. 11969355 10.1006/pmed.2002.1016

[27] West DS, Greene P, Pulley L, Kratt P, Gore S, et al.. (2004) Stepped-care, community clinic interventions to promote mammography use among low-income rural African American women. Health Educ Behav 31(4:Suppl): 29S–44S. 10.1177/1090198104266033 15296690

[28] Kreuter MW, Sugg-Skinner C, Holt CL, Clark EM, Haire-Joshu D, et al (2005) Cultural tailoring for mammography and fruit and vegetable intake among low-income African-American women in urban public health centers. Prev Med 41: 53–62. 15916993 10.1016/j.ypmed.2004.10.013

[29] Powell ME, Carter V, Bonsi E, Johnson G, Williams L, et al.. (2005) Increasing mammography screening among African American women in rural areas. J Health Care Poor Underserv 16 (4:Suppl A): 11–21. 10.1353/hpu.2005.0129 16327093

[30] Champion VL, Springston JK, Zollinger TW, Saywell RM, Monahan PO, et al (2006) Comparison of three interventions to increase mammography screening in low income African American women. Cancer Detect Prev 30: 535–44. 17110056 10.1016/j.cdp.2006.10.003

[31] Russell KM, Champion VL, Monahan PO, Millon-Underwood S, Zhao Q, et al (2010) Randomized trial of a lay health advisor and computer intervention to increase mammography screening in African American women. Cancer Epidemiol Biomarker Prev 19: 201–10. 10.1158/1055-9965.EPI-09-0569 PMC2818428 20056639

[32] Jibaja-Weiss ML, Volk RJ, Kingery P, Smith QW, Holcomb JD (2003) Tailored messages for breast and cervical cancer screening of low-income and minority women using medical records data. Patient Educ Couns 50: 123–32. 12781927 10.1016/s0738-3991(02)00119-2

[33] Nuno T, Martinez ME, Harris R, Garcia F (2011) A Promotora-administered group education intervention to promote breast and cervical cancer screening in a rural community along the U.S.-Mexico border: a randomized controlled trial. Cancer Causes Control 22: 367–74. 21184267 10.1007/s10552-010-9705-4

[34] Maxwell AE, Bastani R, Vida P, Warda US (2003) Results of a randomized trial to increase breast and cervical cancer screening among Filipino American women. Prev Med 37: 102–9. 12855209 10.1016/s0091-7435(03)00088-4

[35] Dietrich AJ, Tobin JN, Cassells A, Robinson C, Greene MA, et al (2006) Telephone care management to improve cancer screening among low-income women: a randomized, controlled trial. Ann Int Med 144: 563–71. 16618953 10.7326/0003-4819-144-8-200604180-00006 PMC3841972

[36] Paskett E, Tatum C, Rushing J, Michielutte R, Bell R, et al (2006) Randomized trial of an intervention to improve mammography utilization among a triracial rural population of women. J Natl Cancer Inst 98: 1226–37. 16954475 10.1093/jnci/djj333 PMC4450352

[37] Kim YH, Sarna L (2004) An intervention to increase mammography use by Korean American women. Oncol Nurs Forum 31: 105–10. 14722594 10.1188/04.ONF.105-110

[38] Denhaerynck K, Lesaffre E, Baele J, Cortebeeck K, Van OE, et al (2003) Mammography screening attendance: meta-analysis of the effect of direct-contact invitation. Am J Prev Med 25: 195–203. 14507525 10.1016/s0749-3797(03)00201-0

[39] Page A, Morrell S, Chiu C, Taylor R, Tewson R (2006) Recruitment to mammography screening: a randomised trial and meta-analysis of invitation letters and telephone calls. Aust N Z J Public Health 30: 111–8. 16681329 10.1111/j.1467-842x.2006.tb00101.x

[40] Bonfill X, Marzo M, Pladevall M, Marti J, Emparanza JI (2001) Strategies for increasing women participation in community breast cancer screening. Cochrane Database of Systematic Reviews 1: CD002943. 10.1002/14651858.CD002943 PMC6457645 11279781

[41] Independent UK Panel on Breast Cancer Screening (2012) The benefits and harms of breast cancer screening: an independent review. Lancet 380: 1778–86. 23117178 10.1016/S0140-6736(12)61611-0

[42] Atri J, Falshaw M, Gregg R, Robson J, Omar RZ, et al (1997) Improving uptake of breast screening in multiethnic populations: a randomised controlled trial using practice reception staff to contact non-attenders. BMJ 315: 1356–9. 9402779 10.1136/bmj.315.7119.1356 PMC2127854

[43] Sutton S, Bickler G, Sancho-Aldridge J, Saidi G (1994) Prospective study of predictors of attendance for breast screening in inner London. J Epidemiol Community Health 48: 65–73. 8138773 10.1136/jech.48.1.65 PMC1059897

[44] Hoare T, Thomas C, Biggs A, Booth M, Bradley S, et al (1994) Can the uptake of breast screening by Asian women be increased? A randomized controlled trial of a linkworker intervention. J Public Health Med 16: 179–185. 7946492 10.1093/oxfordjournals.pubmed.a042954

[45] O'Connor AM, Griffiths CJ, Underwood MR, Eldridge S (1998) Can postal prompts from general practitioners improve the uptake of breast screening? A randomised controlled trial in one east London general practice. J Med Screen 5: 49–52. 9575461 10.1136/jms.5.1.49

[46] Han HR, Lee JE, Kim J, Hedlin HK, Song H, et al (2009) A meta-analysis of interventions to promote mammography among ethnic minority women. Nurs Res 58: 246–54. 19609176 10.1097/NNR.0b013e3181ac0f7f PMC2862462

[47] Egger M, Davey Smith G, Altman DG (2001) Systematic reviews in health care: meta-analysis in context. London: BMJ Books.

[48] Howard M, Agarwal G, Lytwyn A (2009) Accuracy of self-reports of Pap and mammography screening compared to medical record: a meta-analysis. Cancer Causes Control 20: 1–13. 18802779 10.1007/s10552-008-9228-4

[49] Rimer BK (1994) Mammography use in the U.S.: Trends and the impact of interventions. Ann Behav Med 16: 317–326.

[50] Doi SA, Thalib L (2008) A quality-effects model for meta-analysis. Epidemiology 19: 94–100. 18090860 10.1097/EDE.0b013e31815c24e7

[51] Ahmed NU, Haber G, Semenya KA, Hargreaves MK (2010) Randomized controlled trial of mammography intervention in insured very low-income women. Cancer Epidemiol Biomarker Prev 19: 1790–8. 10.1158/1055-9965.EPI-10-0141 20587669

[52] Champion V, Skinner CS, Hui S, Monahan P, Juliar B, et al (2007) The effect of telephone v. print tailoring for mammography adherence. Patient Educ Couns 65: 416–423. 17196358 10.1016/j.pec.2006.09.014 PMC1858664

[53] Dietrich AJ, Tobin JN, Cassells A, Robinson CM, Reh M, et al (2007) Translation of an efficacious cancer-screening intervention to women enrolled in a medicaid managed care organization. Ann Fam Med 5: 320–7. 17664498 10.1370/afm.701 PMC1934974

[54] Mishra SI, Bastani R, Crespi CM, Chang LC, Luce PH, et al (2007) Results of a randomized trial to increase mammogram usage among Samoan women. Cancer Epidemiol Biomarker Prev 16: 2594–2604. 10.1158/1055-9965.EPI-07-0148 PMC3612893 18086763

[55] Oleske DM, Galvez A, Cobleigh MA, Ganschow P, Ayala LD (2007) Are tri-ethnic low-income women with breast cancer effective teachers of the importance of breast cancer screening to their first-degree relatives? Results from a randomized clinical trial. Breast J 13: 19–27. 17214789 10.1111/j.1524-4741.2006.00358.x

[56] Phillips CE, Rothstein JD, Beaver K, Sherman BJ, Freund KM, et al.. (2010) Patient navigation to increase mammography screening among inner city women. J Gen Intern Med DOI: 10.1007/s11606-010-1527–2. 10.1007/s11606-010-1527-2 PMC3019333 20931294

