# Supplementary material for: Interventions to Increase the Uptake of Mammography amongst Low Income Women: A Systematic Review and Meta-Analysis
Source: PLoS One. 2013 Feb 22;8(2):e55574. doi: 10.1371/journal.pone.0055574 (PMC3579869; doi:10.1371/journal.pone.0055574)
Supplement: File S1 — Stratified meta-analyses examining potential sources of heterogeneity for length of follow-up (Figure s1A), source outcome (Figure s1B), location (Figure s1C), whether mammography was free or not (Figure s1D), whether control was usual care or not (Figure s1E) and by level of randomisation (Figure s1F). Testing for small study bias by Funnel plot (Figure s2A) and Egger plot (Figure s2B) and assessing publication bias by stratified meta-analysis (Figure s2C). (DOCX) [file pone.0055574.s001.docx]

**Supplementary Figures**

**Figure s1A** Stratified meta-analysis by length of follow-up for the association between intervention and mammography uptake in low income women.

**Figure s1B** Stratified meta-analysis by source outcome for the association between intervention and mammography uptake in low income women.

**Figure s1C** Stratified meta-analysis by location for the association between intervention and mammography uptake in low income women.

**Figure s1D** Stratified meta-analysis by whether mammography was free or not for the association between intervention and mammography uptake in low income women.

**Figure s1E** Stratified meta-analysis by whether control was usual care or not for the association between intervention and mammography uptake in low income women.

**Figure s1F** Stratified meta-analysis by level of randomisation for the association between intervention and mammography uptake in low income women.

**Figure s2A** Funnel plot to test for small study bias.

**Figure s2B** Egger plot to test for small study bias.

**Figure s2C** Stratified meta-analysis by time lag for the association between intervention and mammography uptake in low income women.

**Figure S1A:** Interventions and mammography stratified by length of follow-up

**Figure S1B:** Interventions and mammography stratified by source outcome

**Figure S1C:** Interventions and mammography stratified by location

**Figure S1D:** Interventions and mammography stratified by free mammography

**Figure S1E:** Intervention and mammography stratified by type of control

**Figure S1F:** Interventions and mammography stratified by level of randomisation

**Figure S2A**

Footnote: Two outliers with RD > 0.4 are from Puschel et al 2010 study

**Figure S2B**

**Figure S2C:** Interventions and mammography stratified by timelag
