# Supplementary material for: Interventions to Increase the Uptake of Mammography amongst Low Income Women: A Systematic Review and Meta-Analysis
Source: PLoS One. 2013 Feb 22;8(2):e55574. doi: 10.1371/journal.pone.0055574 (PMC3579869; doi:10.1371/journal.pone.0055574)
Supplement: Table S1 — Search strategy used to search Medline and Embase databases, to April 2012. (DOCX) [file pone.0055574.s002.docx]

**Table s1: Search strategy used to search Medline and Embase databases, to April 2012**

|  | **Searches** | **Results**  **Medline** | **Results**  **Embase** |
| --- | --- | --- | --- |
| 1 | breast* screen*.tw. | 1461 | 1872 |
| 2 | mammogra*.tw. | 20621 | 25946 |
| 3 | exp Mammography/ | 21936 | 35781 |
| 4 | exp Poverty/ | 26606 | 25624 |
| 5 | exp Socioeconomic Factors/ | 300384 | 148009 |
| 6 | exp Education/ | 563045 | 833922 |
| 7 | depriv*.tw. | 52982 | 60707 |
| 8 | educat*.tw. | 302011 | 372561 |
| 9 | povert*.tw. | 11909 | 12968 |
| 10 | low income.tw. | 13412 | 15312 |
| 11 | exp Randomized Controlled Trials as Topic/ | 79690 | 14797 |
| 12 | (random* adj3 trial*).tw | 139486 | 186752 |
| 13 | random*.tw | 551063 | 716360 |
| 14 | 1 or 2 or 3 | 27707 | 40500 |
| 15 | 4 or 5 or 6 or 7 or 8 or 9 or 10 | 995727 | 1131117 |
| 16 | 11 or 12 or 13 | 590244 | 723956 |
| 17 | 14 and 15 and 16 | 477 | 499 |
| 18 | limit 17 to yr="2002 -Current" | 236 | 271 |
